# Cardio-pulmonary nematodes of the red fox (*Vulpes vulpes*) of Sardinia, Italy

**DOI:** 10.1007/s00436-023-07882-8

**Published:** 2023-05-22

**Authors:** Francesca Nonnis, Claudia Tamponi, Gabriele Tosciri, Maria Manconi, Flavia Pudda, Pierangela Cabras, Giorgia Dessì, Antonio Scala, Antonio Varcasia

**Affiliations:** 1 Parassitologia Veterinaria, Dipartimento di Medicina Veterinaria, Università degli Studi di Sassari, Via Vienna, 2, 07100 Sassari, Italy; 2 CARFS (Centro di Recupero della Fauna Selvatica Ferita) Forestas, Bonassai, Sardinia Italy; 3 IZS (Istituto Zooprofilattico Sperimentale) Sardegna, Tortoli, Sardinia Italy

**Keywords:** Wildlife, Lungworms, Necropsy, Morphological identification, Molecular analysis

## Abstract

Cardio-pulmonary parasites, such as *Angiostrongylus vasorum*, *Crenosoma vulpis*, and *Eucoleus aerophilus*, pose a significant concern on account of pulmonary and cardiac problems they induce in dogs. While the red fox is known to be a key reservoir host for *A. vasorum* and may also play a role in transmitting *C. vulpis* and *E. aerophilus*, there has been no recent research on these parasites in foxes from Sardinia, with the most current studies dating back to 1986. A survey was conducted on red foxes in Sardinia, where a total of 51 foxes were collected, necropsied, and examined for adult worms in their hearts and lungs. The worms were identified using morphometric analysis and molecular methods. The results showed a 54.9% overall prevalence at dissection: 45.1% of the foxes were positive for *E. aerophilus*, 17.6% for *C. vulpis*, and 13.7% for *A. vasorum*. The molecular analyses validated the morphological characterization. In comparison to previous research, which found 13 out of 85 foxes to be positive for *A. vasorum* with a prevalence rate of 15.3% and 1 for *E. aerophilus* with a prevalence of 1.2%, this study showed an increased prevalence of *E. aerophilus* and *C. vulpis*, and a decrease in the prevalence of *A. vasorum*. These results indicate that the red foxes in Sardinia represent a reservoir host for cardio-pulmonary nematodes and it should be considered in the differential diagnosis of respiratory distress syndrome in dogs.

## Introduction

Cardio-pulmonary nematodes, such as *Angiostrongylus vasorum*, *Crenosoma vulpis*, and *Eucoleus aerophilus*, are of major concern for the scientific and veterinary communities. These parasites are responsible for a wide range of clinical manifestations, especially in domestic dogs. *Angiostrongylus vasorum* and *C. vulpis* have an indirect life cycle involving gastropod mollusks as intermediate hosts. They usually infect red foxes and domestic dogs, but their presence has also been reported in other wild canids and mustelids (Tieri et al. 2021; Simpson et al. 2016; Martínez-Rondán et al. 2019; Latrofa et al. 2015). On the other hand, *Eucoleus aerophilus* has a direct life cycle, with earthworms serving as paratenic host (Anderson 2000), and it has a cosmopolitan distribution affecting both domestic and wild mammal, including humans, often mimicking bronchial carcinoma with cough, mucoid sputum, fever, and dyspnea (Lalošević et al. 2008).

These nematodes cause a range of symptoms in domestic animals. Respiratory symptoms can range from mild, such as coughing, sneezing, dyspnea, or exercise intolerance, to severe, such as pulmonary hypertension and cor pulmonale in dogs infected with *A. vasorum* (known as “the French heartworm”), which may lead sometimes to fatal cardio-pulmonary disease (Conboy 2009; Koch et al. 2009). In addition, canine angiostrongylosis can also result in different clinical manifestations, such as bleeding disorders and coagulopathies. These disorders are thought to be caused by disseminated intravascular coagulation and consumptive coagulopathy, and may result in subcutaneous hematomas, ecchymotic hemorrhages in the conjunctival, episcleral, and gingival areas, and occasionally fatal cerebral, spinal, or abdominal hemorrhage. Other signs, such as ascites, syncope, vomiting, and signs of central nervous system disorders, may also be observed (Conboy 2009; Chapman et al. 2004). The clinical signs and outcomes of these infections vary depending on parasite localization and burden, as well as host’s age, immune response, and the presence of concurrent infections in the host (Conboy 2009). The contact between domestic and wild animals may represent a key factor in the epidemiology of this parasitosis, as wild animals serve as a significant reservoir for several cardio-pulmonary species (Otranto et al. 2015).

The red fox (*Vulpes vulpes*) is a highly adaptable wild canid that is widely distributed across the world. It has been recognized as a key player in the transmission of various parasites (Otranto et al. 2015) due to its ability to thrive in both rural and urban habitats (Deplazes et al. 2004). Furthermore, the fox is a generalist predator that feeds on a wide variety of preys, resulting in high risk of infection by ingestion of both intermediate and paratenic hosts of various parasites (Bolt et al. 1994).

Red foxes are abundant in Sardinia, and they are widespread throughout the island. However, the possible involvement of this wild canid as a reservoir for these parasites has not been fully understood on the island, and indeed, the last survey carried out in Sardinia dates to 1986, where 85 foxes collected from 13 sites in the island were necropsied, and hearts and lungs dissected (Leoni et al. 1986).

Given the spread of these infections across Europe and their clinical relevance in pets, the present study aimed to determine the potential role of the red fox as a reservoir for cardio-pulmonary nematodes in Sardinia, to gain a better understanding of its implication in the transmission to domestic animals.

## Material and methods

Between 2015 and 2021, 51 red foxes, found dead in road accidents, were collected from all regions of Sardinia. Animals were transported in sealed plastic bags to the Department of Veterinary Medicine of the University of Sassari, Italy. Data on sex and origin were recorded, and a unique identification number was assigned to each animal. Age was estimated based on body size and dentition, according to Roulichova and Andera (2007). Afterwards, carcasses were stored at −80 °C to inactivate potential zoonotic agents and then kept at −20 °C until examination.

At necropsy, hearts and lungs were collected. Hearts were separated from lungs, and heart chambers and large blood vessels were dissected and examined for adult worms under a stereomicroscope. The lungs were thoroughly examined externally before proceeding with the longitudinal dissection of the trachea and bronchial tree. Adult worms collected from the heart and lungs were washed in saline solution and stored in 70% ethanol. Parasites were individually clarified with 1:1 glycerol-ethanol and then placed on slides and observed under the light microscope. Images and measurements were taken by using a digital image processing system (Olympus BX41; Soft Imaging solution GMBH LG20, Munster, Germany). They were then sexed and identified at the species level using morphometrical keys available in the primary literature (Anderson 2000).

In addition to morphological identification, three representative samples of each species (*A. vasorum*, *C. vulpis*, and *E. aerophilus*) were molecularly characterized through PCR using NC1 and NC2 primers for the amplification of the rRNA region of ITS-2 and partial regions of 5.8S and 28S rRNA (Gasser et al. 1993). The nucleotide sequences (480 bp) were analyzed through FinchTV viewer (Geospiza, Seattle, WA, USA). The reference sequences for each species were retrieved through BLAST algorithm (https://blast.ncbi.nlm.nih.gov/Blast.cgi) based on a sequence similarity threshold of >99%, followed by multiple alignment using MEGA X (Kumar et al. 2018).

## Results and discussion

A total of 51 red foxes were examined, of which 36 were males and 15 females. Concerning age, 34 red foxes were categorized as adults (≥12 months) and 17 as subadults (<12 months). An overall prevalence of 54.9% (95%CI: 41.2–68.6) was detected, with 28 animals harboring at least one parasite species at dissection of lungs and heart. The morphological examination of recovered adult specimens revealed three different species: *E. aerophilus* (45.1%; 95%CI: 31.4–58.7), *C. vulpis* (17.6%; 95%CI: 7.2–28.1), and *A. vasorum* (13.7%; 95%CI: 4.3–23.2).

In 13.7% (95%CI: 4.3–23.2) of the animals, a concurrent infection was found: 5.9% of the foxes were co-infected by *A. vasorum*, *C. vulpis*, and *E. aerophilus*, 3.9% by *C. vulpis* and *E. aerophilus*, and 3.9% by *A. vasorum* and *E. aerophilus*.

Prevalence was higher in males (63.9%; 95%CI: 48.2–79.6) than in females (33.3%; 95%CI: 9.5–57.2), although the difference was not statistically significant (χ^2^ = 2.8539; *P* = 0.0912). Likewise, no correlation was observed between infection and age of the animal (χ^2^ = 0.6335; *P* = 0.4261), with only a slight increase in prevalence recorded in adults (58.8%; 95%CI: 42.3–75.4) compared to young individuals (47.1%; 95%CI: 23.3–70.8) (Table 1).

**Table 1:** Prevalence of adult specimens of *E. aerophilus*, *C. vulpis*, and *A. vasorum* observed at dissection, reported per sex and age class of the animals

Animal category	Examined foxes (no.)	No. of positives	Overall prevalence (%)	CI	*E. aerophilus*			*C. vulpis*			*A. vasorum*		
					No.	*P* (%)	CI	No.	*P* (%)	CI	No.	*P* (%)	CI
Males	36	23	63.9%*	48.2–79.6	18	50%	33.7–66.3	9	25%	10.8–39.1	6	16.7%	4.5–28.8
Females	15	5	33.3%*	9.5–57.2	5	33.3%	9.5–57.2	0	0%		1	6.7%	−6.0–19.3
<12 months	17	8	47.1%**	23.3–70.8	7	41.2%	17.8–64.6	3	17.6%	−0.5–35.8	1	5.9%	−5.3–17.1
≥12 months	34	20	58.8%**	42.3–75.4	16	47.1%	30.3–63.8	6	17.6%	4.8–30.5	6	17.6%	4.8–30.5
Total	51	28	54.9%	41.2–68.6	23	45.1%	31.4–58.7	9	17.6%	7.2–28.1	7	13.7%	4.3–23.2

*CI* 95% confidence interval

*χ^2^ = 2.8539; *P* value = 0.0912

**χ^2^ = 0.6335; *P* value = 0.4261

A total of 107 *E. aerophilus*, 67 *C. vulpis*, and 24 *A. vasorum* specimens were collected, with a mean intensity (mI) of 4.6 for *E. aerophilus*, 7.4 for *C. vulpis*, and 3.4 for *A. vasorum*.

The molecular analysis corroborated the morphological characterization, and it was confirmed through BLAST analysis that three species of cardio-pulmonary nematodes affect red foxes of Sardinia. The nucleotide sequences for *A. vasorum* had 100% similarity with the sequences from the UK (GU045374) and Portugal (EU627595). Sequence-based identification revealed that the samples of *C. vulpis* were 100% identical to the sequences from the UK (MT808324) and USA (OM480716). ITS-2 and partial regions of the 5.8S and 28S rRNA sequences for *E. aerophilus* had a matching sequence identity of 100% with a sequence from Australia (MW709573) and 99.80% with a sequence from Germany (MF599385).

This study represents the first epidemiological survey on lungworms in red foxes of Sardinia since the one conducted by Leoni et al. (1986). Its purpose is to offer up-to-date information on the status of infection in foxes with these parasites and evaluate the role of red foxes as reservoir hosts for domestic animals. The results of the present investigation report the occurrence of three major cardio-pulmonary nematodes in this wild canid, indicating that their presence in Sardinia may have substantial epidemiological implications.

The prevalence observed in the current study differed from that previously documented in Leoni et al. (1986), particularly regarding *E. aerophilus*, that in the previous study were found in only one of the 85 animals tested (1.2%) while the prevalence (45.1%) in the current study was found to be significantly higher (χ^2^ = 39.34; *P* < 0.0001). The prevalence of *A. vasorum* (13.7%) appeared to have slightly decreased from 1986 (15.3%; 13/85) to date (13.7%; 7/51) although this difference was not statistically significant (χ^2^ = 0.06; *P* = 0.802). *Crenosoma vulpis* was not detected at all in the previous study (Leoni et al. 1986).

Over the past decade the expansion of cardio-pulmonary infections has led to an increase in concern among vet practitioners and pet owners. Moreover, considering that the red fox is an important source of zoonotic parasites that can cause life-threatening diseases, such as *Echinococcus multilocularis*, *Toxocara canis*, and *Trichinella* spp., we should exercise extreme caution in areas where domestic and wild animals coexist (Lazar et al. 2006; Deplazes et al. 2004).

For this reason, a similar investigation on the presence of gastrointestinal parasites in the organs collected from the same foxes is currently underway to acquire a more comprehensive understanding of their role in these parasitoses.

In the last years, Sardinia has captured the interest of the scientific community, due to the high prevalence of bronchopulmonary nematodes observed in pets (Tamponi et al. 2014; Pipia et al. 2014). Several species have been described to date, including *Angiostrongylus chabaudi*, found for the first time in a domestic cat after its first description in 1957 in wildcats from central Italy (Varcasia et al. 2014). The potential role of other wild carnivores (as wild cats and pine martens) as reservoirs of these parasites has not been fully elucidated; hence, additional research is required. In fact, gaining fresh insights into dynamics of infection in wild animals and the factors underlying the spillover to pets is of utmost significance.

## Data Availability

The datasets used and/or analyzed during the current study are available from the corresponding author on reasonable request.
