# Correction to: Cardio-pulmonary nematodes of the red fox (*Vulpes vulpes*) of Sardinia, Italy

**DOI:** 10.1007/s00436-023-07897-1

**Published:** 2023-06-14

**Authors:** Francesca Nonnis, Claudia Tamponi, Gabriele Tosciri, Maria Manconi, Flavia Pudda, Pierangela Cabras, Giorgia Dessì, Antonio Scala, Antonio Varcasia

**Affiliations:** 1 Parassitologia Veterinaria, Dipartimento di Medicina Veterinaria, Università degli Studi di Sassari, Via Vienna, 2, 07100 Sassari, Italy; 2 CARFS (Centro di Recupero della Fauna Selvatica Ferita) Forestas, Bonassai, Sardinia Italy; 3 IZS (Istituto Zooprofilattico Sperimentale) Sardegna, Tortoli, Sardinia Italy

**Correction to: Parasitology Research**

10.1007/s00436-023-07882-8

The authors regret that the appearance of their first and last names in the original article were swapped. Their correct order appears in this erratum article.

The original article has been corrected.

